# The Grafting of Universal T-Helper Epitopes Enhances Immunogenicity of HIV-1 Tat Concurrently Improving Its Safety Profile

**DOI:** 10.1371/journal.pone.0114155

**Published:** 2014-12-22

**Authors:** Venkatesh P. Kashi, Rajesh A. Jacob, Raghavendra A. Shamanna, Malini Menon, Anangi Balasiddaiah, Rebu K. Varghese, Mahesh Bachu, Udaykumar Ranga

**Affiliations:** 1 HIV/AIDS laboratory, Molecular Biology and Genetics Unit, Jawaharlal Nehru Center for Advanced Scientific Research, Bangalore, 560064, India; 2 Cellular Immunology Group, International Center for Genetic Engineering and Biotechnology, Cape Town, South Africa; 3 Laboratory of Molecular Gerontology, National Institute on Aging, Baltimore, Maryland, United States of America; University of South Carolina School of Medicine, United States of America

## Abstract

Extracellular Tat (eTat) plays an important role in HIV-1 pathogenesis. The presence of anti-Tat antibodies is negatively correlated with disease progression, hence making Tat a potential vaccine candidate. The cytotoxicity and moderate immunogenicity of Tat however remain impediments for developing Tat-based vaccines. Here, we report a novel strategy to concurrently enhance the immunogenicity and safety profile of Tat. The grafting of universal helper T-lymphocyte (HTL) epitopes, Pan DR Epitope (PADRE) and Pol_711_ into the cysteine rich domain (CRD) and the basic domain (BD) abolished the transactivation potential of the Tat protein. The HTL-Tat proteins elicited a significantly higher titer of antibodies as compared to the wild-type Tat in BALB/c mice. While the N-terminal epitope remained immunodominant in HTL-Tat immunizations, an additional epitope in exon-2 was recognized with comparable magnitude suggesting a broader immune recognition. Additionally, the HTL-Tat proteins induced cross-reactive antibodies of high avidity that efficiently neutralized exogenous Tat, thus blocking the activation of a Tat-defective provirus. With advantages such as presentation of multiple B-cell epitopes, enhanced antibody response and importantly, transactivation-deficient Tat protein, this approach has potential application for the generation of Tat-based HIV/AIDS vaccines.

## Introduction

Transactivator of transcription (Tat) of HIV-1 is essential for the viral gene expression and infectivity [1]–[3]. Nearly two-thirds of Tat made by infected CD4+ T-cells are secreted into the extra-cellular milieu [4] and the extracellular Tat (eTat) can be taken up by cells. Subsequently, Tat can enter the nucleus and regulate several host genes that can impact the immune system [5]. In addition, Tat can contribute to the viral pathogenesis by activating latent viral reservoirs [6]. Neutralization of eTat therefore could be an important objective, making Tat a potential vaccine candidate.

Tat offers several advantages as a candidate antigen. Most importantly, humoral and cell-mediated immune responses to Tat protect subjects from disease progression [7]–[14]. Vaccine studies with Tat [15], [16], recombinant vaccinia virus expressing Tat and Rev [17] and rhesus cytomegalovirus vectors expressing Tat protect macaques against the viral challenge [18]. A pilot study showed that an HIV vaccine based on both the Tat and Env proteins could efficiently control an intrarectal Simian-human immunodeficiency virus (SHIV) challenge [19]. Studies suggest that Tat-gp120 interaction facilitates viral entry into cells [18], [20] and interfering with this interaction can be a potential avenue for HIV vaccines.

Despite the advantages, certain limitations of Tat restrict its application as a vaccine for HIV/AIDS. Only a small fraction of the seropositive subjects makes anti-Tat antibodies [18] with even fewer showing isotype switch to IgG which suggests lack of efficient T-help [21]. Immunization with a cocktail of Tat peptides failed to protect rhesus macaques against the mucosal challenge with SHIV [22]. Tat expressed by a replication defective adenovirus 5 was ineffective against an intravenous viral challenge [23]. Several immunizations with the Tat toxoid [24], but not fewer [25], were required to elicit a protective immune response in macaques against an intravenous SHIV89.6D challenge. Studies show that Tat is an immunosuppressive agent [26] and can induce apoptosis of immune cells [27], although, contradictory studies also exist [28], [29]. While the varying experimental conditions could partly explain the discordant results, the intrinsic moderate immunogenicity of Tat may be an important reason for these findings.

In this study, we describe a novel strategy to boost the antibody response against Tat and simultaneously abrogate its transactivation potential. We grafted two different universal helper T-lymphocyte (HTL) epitopes, pan-DR epitope (PADRE) and Pol_711_ to disrupt the cysteine-rich domain (CRD) and/or the basic domain (BD). We demonstrate that HTL-Tat protein immunizations elicit qualitatively and quantitatively superior antibody responses in mice. Importantly, the HTL-Tat proteins are deficient in the transactivation potential therefore making them safer for vaccine studies.

## Materials and Methods

### Tat-expression vectors

**Figure 1:**
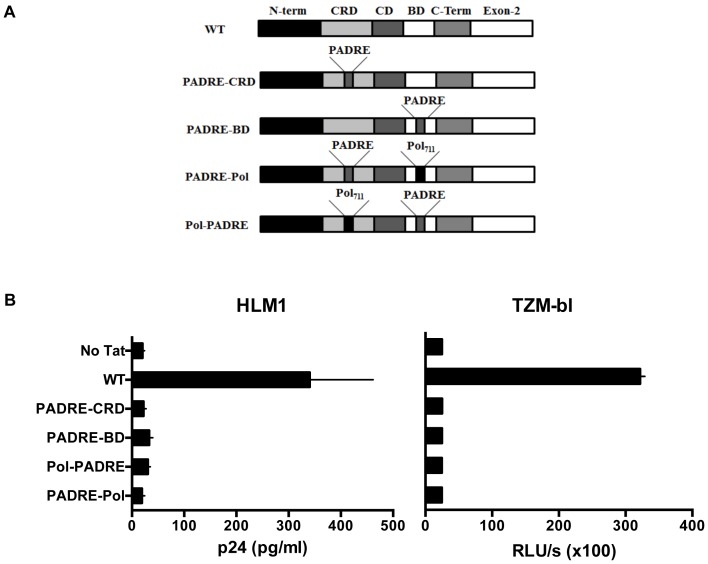
HTL-Tat proteins are transactivation deficient. (A) Domain structures of the five Tat constructs are illustrated. WT: wild type, N-term: amino terminal region, CRD: Cystine-rich domain, CD: core domain, BD: basic domain and C-term: carboxy terminal of exon-1 (B) The HTL-Tat proteins fail to transactivate in HLM1 (left panel) or TZM-bl (right panel) cells. The data for the 72 h time point are presented. The x-axis represents the mean amount of p24 or luciferase + SD and the y-axis the Tat construct used. The experiments were performed in biological triplicates and the plotted data are representative of two independent experiments.

All the Tat vectors were based on the plasmid pET21b+ (Novagen). The construction of the wild-type Tat (WT-Tat) vector from a primary subtype C clinical isolate was described previously [30]. Using overlap PCR, we grafted PADRE (AKFVAAWTLKAAA) and Pol_711_ (EKVYLAWVPAHKGIG) coding sequences into the CRD and/or BD of Tat. In the CRD, the epitopes were cloned between residues C30 and S31 and in the BD between K52 and R53. Two vectors containing the PADRE insertion in the CRD and BD (PADRE-CRD and PADRE-BD) were constructed first. The dual-HTL Tat vectors PADRE-Pol and Pol-PADRE were constructed by subsequent grafting of the Pol-epitope into the PADRE-CRD and PADRE-BD single-HTL vectors, respectively. The oligonucleotides used for the construction of these vectors and Tat-domains into which the HTL-epitopes were grafted have been summarized in S1 Table. In Fig. 1A, an illustration of the domain structure of Tat constructs is shown.

### Immunization protocol

The Institutional Animal Ethics Committee of Jawaharlal Nehru Center for Advanced Scientific Research (JNCASR) approved all the experimental work following the guidelines stipulated by The Committee for the Purpose of Control and Supervision of Experiments on Small Animals, Government of India (201/CPCSEA). All mice were housed in ventilated cages under standard conditions (23°C with 12 h light/dark cycle) with easy access to food and water. Care was taken to minimize stress to the mice during experimental procedures. Recombinant proteins were expressed and purified as described previously [30]. Monomeric Tat protein was gel-purified following SDS-PAGE separation and used for immunizations. The proteins were emulsified with complete Freund's adjuvant (CFA) for the priming and with incomplete Freund's adjuvant (IFA) for the booster immunizations. Six-week-old female BALB/c mice procured from the JNCASR animal facility were immunized subcutaneously.

### Enzyme linked immunosorbent assay

Tat-antigen coating into microtiter plates, blocking, washing and substrate solutions were as described previously [21]. Serially diluted sera were added and the plates were incubated at 37°C for 1 h and subsequently anti-mouse HRPO (Calbiochem) was added. After 1 h incubation, substrate solution was added and the plates were incubated in the dark. Enzyme reaction was stopped with 1N HCl. Absorbance was measured using a microplate reader (Bio-Rad) and antibody titers were calculated by endpoint dilution. For the antibody isotype analysis, rabbit secondary antibodies specific to mouse IgG1 or IgG2a (Sigma-Aldrich) were used. Following which, anti-rabbit HRPO was added to each well and the subsequent steps were as mentioned above. For the cross-reactivity assay, Tat from clades A (p92UG037), B (pYU2), C (pE-TatBL43.CS) and D (p84ZR085) were used as antigens. Epitope mapping was carried out using peptides listed in S2 Table. Avidity assay was carried out as previously described with modifications [21]. One microgram of the peptide encompassing 1–20 amino acids of Tat was used as the antigen.

### Lymphoproliferation assay

Splenocytes from the immunized mice were stained with carboxyfluoresceinsuccinimidyl ester (CFSE) as described before [31]. Appropriate peptides (5 µg/ml) were added to cells plated in complete RPMI medium in 96-well plates and harvested after 5 days, washed with PBS and stained with anti-CD4-PE antibody (Southern Biotech). Phytohemagglutinin (PHA) was used as the positive control. Proliferation data was acquired on FACSCalibur (BD Biosciences) and CFSE-dilution in live CD4+ cells was analyzed using FlowJo (Tree Star).

### Tat transactivation assay

Transactivation assay was carried out in TZM-bl and HLM1 cells (obtained from AIDS Research and Reference Program). TZM-bl cells plated in plain DMEM supplemented with 5 µg/ml of highly purified Tat proteins were incubated for 2 h, washed and complete medium was added and incubated for 72 h. Luciferase activity was measured using Bright-Glo Luciferase Assay (Promega) on a SpectraMax L Luminescence reader (Molecular Devices Inc.). The HLM1 cells were incubated with Tat as mentioned above and the culture supernatant was sampled every 24 h. Following viral inactivation with 0.1% Empigen and incubating at 56°C for 30 min, the quantity of p24 in the samples was measured as per manufacturer's instructions (Perkin Elmer Life Sciences).

### Tat-neutralization assay

HLM1 cells were cultured overnight in a 96-well plate. The sera were diluted 1000-fold in serum-free medium, Tat protein (500 ng) was added to 100 µl of diluted serum and the samples were incubated at 37°C for 30 min. The samples were then added to the cells and the plates were incubated for 3 h. The supernatant was removed and complete medium was added. The plates were incubated for 72 h and the levels of p24 in the medium were determined as before. An in-house generated IgG1 monoclonal antibody against C-Tat, E.6.4, was used as neutralization positive control.

### Statistical methods

GraphPad Prism 6 was used to calculate P-values applying Student's *t*-test at 95% confidence interval. P≤0.05 was considered significant.

## Results

### Design of Tat expression vectors

The molecular integrity of the CRD and the BD is critical for Tat function [32], [33]. We therefore hypothesized that disruption of the structural integrity of one or both of these domains, by inserting heterologous amino acid sequences, should abrogate the transactivation function. We grafted two different universal helper T-lymphocyte (HTL) epitopes PADRE and Pol_711_ to disrupt the CRD and/or BD of Tat. We generated four different HTL-Tat bacterial expression vectors (Fig. 1A). In two of the HTL-Tat vectors, the PADRE sequence was inserted into CRD or the BD. In the other two vectors, both the domains were disrupted using the PADRE and the Pol_711_ sequences in two different combinations. In addition to rendering Tat transactivation-deficient, we expected the insertion of HTL-epitopes to provide T-help to antigen-specific B-cells and thus enhance the immunogenicity of Tat.

### Engineering of HTL-epitopes abolishes the transactivation potential of Tat

The transactivation potential of the Tat proteins was examined using two different cell lines. While p24 was used as the reporter for HLM1 cells, luciferase driven by the LTR was used in case of TZM-bl. The exogenously added WT-Tat protein efficiently induced the expression of the viral antigen p24 (Fig. 1B, left panel) or that of the reporter luciferase gene (Fig. 1B, right panel) from the HLM1 and TZM-bl cells, respectively. In contrast, none of the four HTL-Tat proteins was competent in transactivating the viral promoter in either of the cell lines. Importantly, disruption of either CRD or BD was sufficient to abolish the transactivation property of Tat.

### HTL-grafting augments anti-Tat immune responses

**Figure 2:**
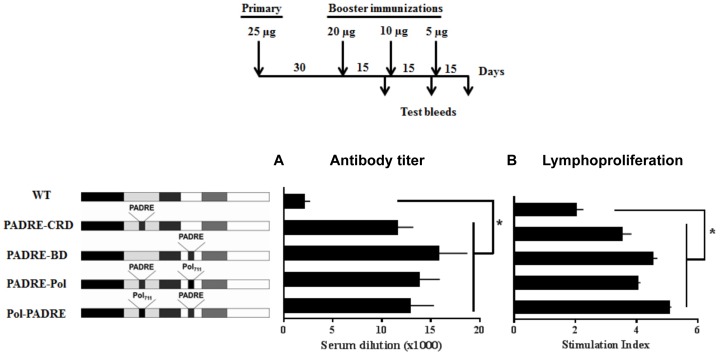
The HTL-Tat proteins elicit a strong immune response. The immunization protocol is represented in the inset schema. (A) Antisera collected 14 days after the final booster were used for the titer determination. X-axis corresponds to antibody titer and y-axis to the Tat protein used in immunizations. (B) Splenocytes harvested from mice 14 days after the final booster immunization were stained with CFSE and dilution of the dye in CD4+ cells was evaluated and the mean stimulation index (SI) + SD was plotted on the x-axis. SI  =  Percent CFSE-low (Peptide-stimulated cells)/Percent CFSE-low (DMSO-treated cells). The data presented are representative of two independent experiments (n = 10, * P<0.05).

The antibody-titers in sera isolated 14 days after the final booster immunizations were determined using ELISA. All the four HTL-Tat proteins elicited significantly higher titer of antigen-specific antibodies as compared to WT-Tat (Fig. 2A). For instance, the PADRE-CRD Tat immunization showed an antibody titer of 11500+/−1640 as compared to that of the WT-Tat, 2200+/−330. The immune response was not significantly different between the single- (PADRE-CRD and PADRE-BD Tat) or dual-HTL-Tat proteins (PADRE-Pol and Pol-PADRE Tat) suggesting that a single HTL-epitope insertion was sufficient to enhance the antibody response.

Next, to confirm the enhanced T-helper response in HTL-Tat, we performed a CFSE-based lymphoproliferation assay using splenocytes isolated from the immunized mice. All the HTL-Tat proteins induced a significantly higher level of CD4+ T-cell proliferation as compared to WT-Tat suggesting that HTL-grafting boosted the T-helper immune response. For instance, PADRE-CRD showed a stimulation index of 3.8+/−0.3 as compared to that of WT- Tat, 1.92+/−0.39 (Fig. 2B and S1 Fig.).

**Figure 3:**
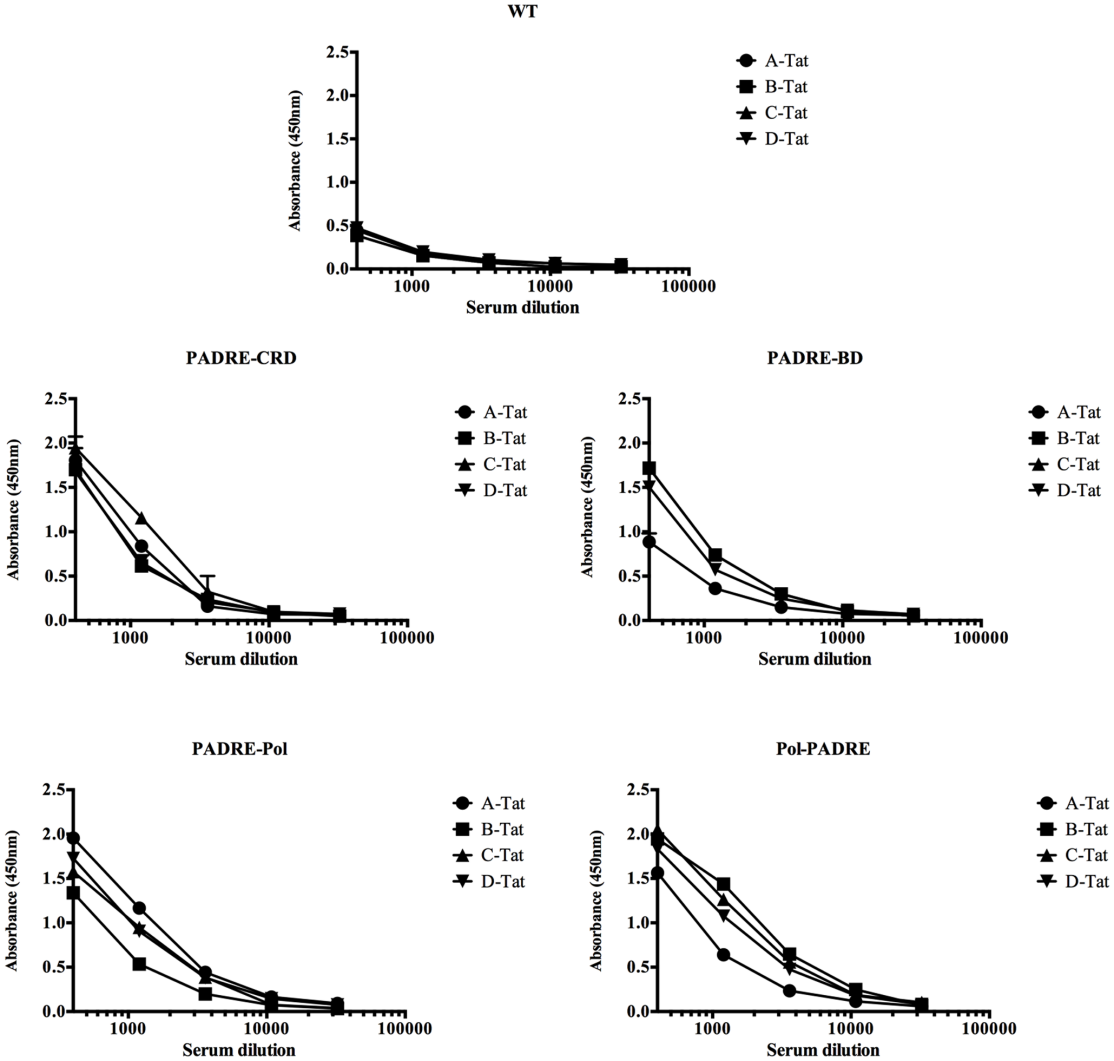
Cross-clade reactivity of the anti-Tat antibodies. The antibody titers were determined by incubating serially diluted antisera in triplicate microtiter wells coated with recombinant Tat from subtypes A, B, C and D. The x-axis represents the serum dilution and the y-axis to mean absorbance + SD. Representative data of two or more independent experiments are shown.

To test the cross-clade reactivity of Tat-antibodies, antisera diluted serially were incubated in micro-titer wells coated with the Tat from HIV subtypes A, B, C and D. Despite the variation in the sequence among the viral subtypes the antibodies raised against the WT-Tat and the HTL-Tat proteins reacted with the heterologous Tat proteins efficiently (Fig. 3). As before, the HTL-Tat induced antibodies demonstrated higher titers as compared to those induced by WT-Tat.

### HTL-Tat immunization spreads the immune response to sub-dominant B-cell epitopes

**Figure 4:**
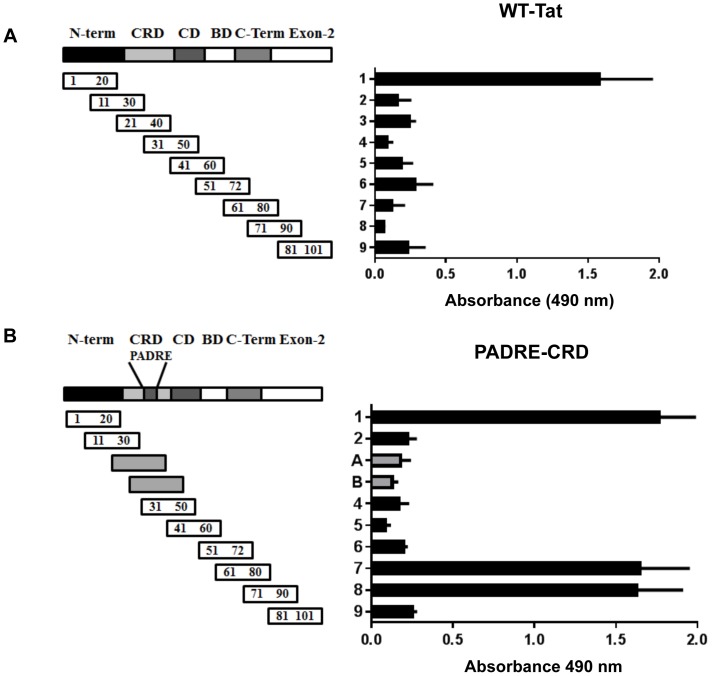
Mapping of the B-cell epitopes in Tat. The antisera collected from mice immunized with (A) WT-Tat or (B) PADRE-CRD proteins were diluted 500 and 1,000 times, respectively, and used in the pepscan analysis. Twenty-mer synthetic peptides with a 10-residue overlap and encompassing the full-length C-Tat consensus sequence were used in the assay. Peptides 1-9 represent full-length subtype C Tat protein. The gray bars (A and B) represent the amino acid sequences generated due to the grafting of the HTL in Tat. The data are presented as the mean absorbance + SD on the x-axis with corresponding peptides on the y-axis. See Figure S2 for data pertaining to the other three HTL-Tat antisera.

Strong T-help facilitates antibody epitope spreading to moderately immunogenic B-cell epitopes. Since we engineered universal HTL-epitopes, we next addressed if the mice elicited antibody response to multiple epitopes. The immunization experiments with WT-Tat protein confirmed the immunodominant nature of the N-terminal epitope (NTE) that has previously been reported (Fig. 4A). Remarkably, mice immunized with PADRE-CRD not only elicited a robust response to NTE, but also to the epitope in the exon-2 of Tat (Fig. 4B). Furthermore, the antibody response to all the epitopes was of similar magnitude. Immunizations with other three HTL-Tat proteins elicited a similar response (S2 Fig.).

### HTL-Tat immunization boosts both Th1 and Th2 responses

**Figure 5:**
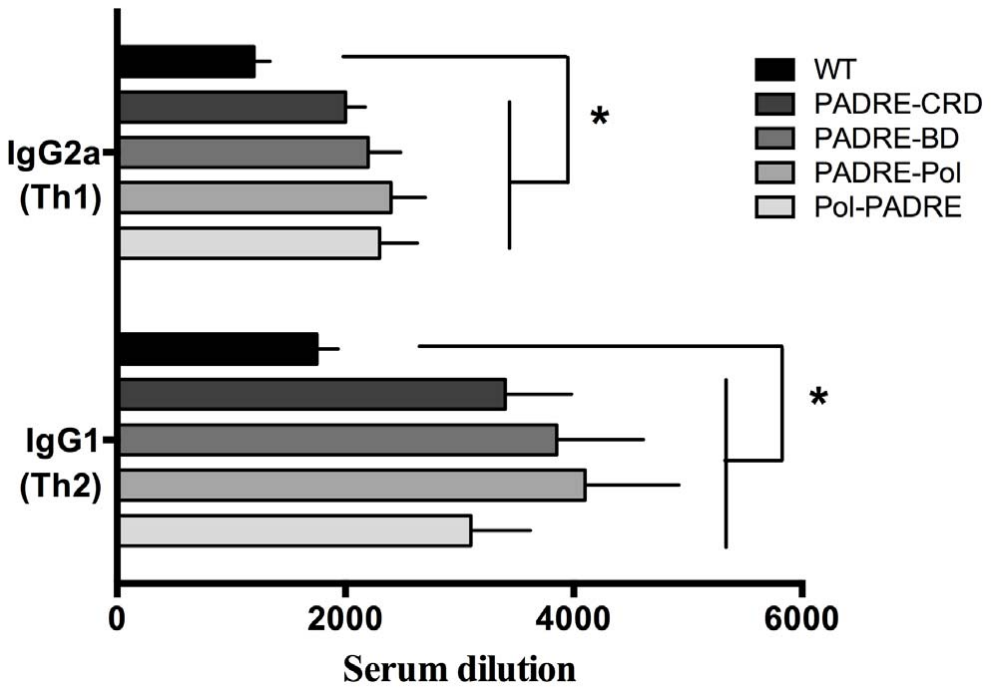
IgG1 and IgG2a profile of anti-Tat antibodies. The isotype profile of the Tat-specific antibodies was determined using IgG2a (Th1) and IgG1- (Th2) specific secondary antibodies. Each bar corresponds to the antisera used in the assay as enlisted in the figure key and are grouped into two based on the isotype-specific secondary antibody used shown on the y-axis and x-axis corresponds to antibody titers. Representative data of two independent experiments are shown. *P<0.05.

Typically in mice, IgG2a and IgG1 correlate with the Th1 and Th2 type of immune responses, respectively. Using IgG2a and IgG1-specific secondary antibodies, we examined the Th-profile of the immune responses following Tat-immunization. Regardless of the Tat protein used, all the immunizations resulted in a mixed Th-profile as both IgG1 and IgG2a antibodies were detected with a predominance of IgG1 (Fig. 5). Notably, the immunization with HTL-Tat enhanced both Th1 and Th2 antibodies despite an expected predisposition to Th2 in BALB/c mice. These data suggest that insertion of HTL-epitopes in Tat resulted in enhancing both the Th1 and Th2 responses in BALB/c mice.

### HTL-grafting induces antibodies of higher avidity

**Figure 6:**
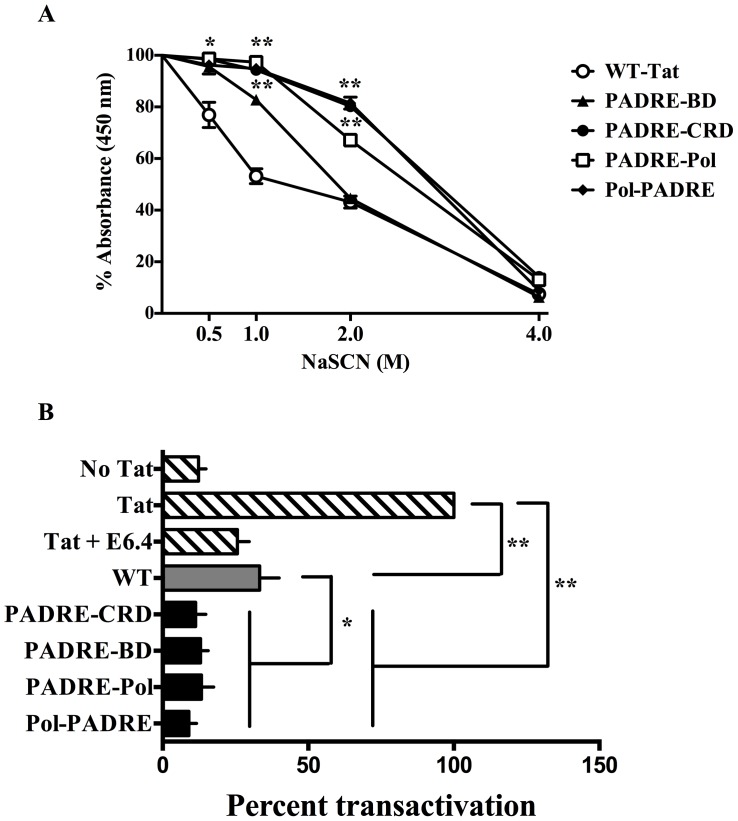
Efficient neutralization of Tat by high avidity antibodies. (A) The HTL-Tat proteins induce the generation of high avidity antisera. Antisera diluted 500-fold were incubated in microtiter wells coated with the amino-terminal peptide (1-20 amino acids) of Tat. Percent absorbance of the NaSCN treated wells was calculated considering the absorbance of the control wells as 100%. The mean percent absorbance + SD values are shown on the y-axis and the concentration of NaSCN on the x-axis. (B) The HTL-Tat antisera block the exogenous Tat efficiently. Percent transactivation was calculated considering the amount of p24 secreted by HLM1 cells in the absence of a neutralizing antibody as 100%. *P<0.05 and **P<0.005.

The avidity of anti-Tat antibodies was determined using sodium thiocyanate (NaSCN), a mild chaotropic reagent. Since all the proteins elicited antibodies against the NTE, here we used a peptide comprising this epitope as the antigen instead of full-length Tat. As the concentration of NaSCN increased from 0.5 to 4.0 M, a larger magnitude of bound antibodies of all the antisera dissociated from the immobilized antigen (Fig. 6A). A significant difference between WT-Tat sera and HTL-Tat was evident at the NaSCN concentrations of 0.5, 1.0 and 2.0 M. The WT-Tat sera showed a decrease of 25% and 50% in absorbance at 0.5 and 1 M NaSCN, respectively, whereas the HTL-Tat sera showed appreciable decrease in absorbance only when incubated with 2 M NaSCN. Upon addition of 4 M NaSCN, all the antibodies ceased to interact with the antigen. The data collectively suggest that HTL-Tat immunizations induced antibodies of higher avidity.

### Tat-antibodies neutralize extracellular Tat efficiently

Finally, we determined the Tat-blocking potential of the antibodies. The recombinant subtype C Tat protein was incubated with Tat-antisera before adding to the culture wells containing HLM1 cells. The amount of p24 in the medium in the absence of any antibody was considered as 100% transactivation. A Tat-specific monoclonal antibody, E6.4, which blocks 75% of the transactivation, was included as a positive control for Tat-neutralization (Fig. 6B). Tat-neutralization potential of the HTL-Tat antisera was significantly superior to that of the WT-Tat (P<0.05). While up to 70% of reduction was seen in the Tat-transactivation in the presence of the WT-Tat antiserum, Tat naturalization was nearly complete in the presence of HTL-Tat antisera.

## Discussion

Tat protein of HIV along with its intracellular function as a transactivator has several pleiotropic and deleterious functions as an extracellular protein. Tat has been shown to up regulate CCR5 and CD25 along with other genes that promote viral infection. In addition, Tat down regulates MHC class I and hence hindering immune surveillance [5]. Furthermore, Tat has been shown to have subtype-specific variations, in that, subtype C Tat induces the expression of immune suppressive IL-10 and IL-4 whereas subtype B Tat enhances pro-inflammatory IL-6 and TNFα [2]. These complications along with the inherent moderate immunogenicity of Tat are a significant challenge for developing Tat-based vaccines. In the present study, we have described a novel strategy of Tat-engineering to generate safe proteins that elicit strong antibody response to multiple epitopes. The CRD and BD of Tat are of significant importance as the loss of integrity of either of these domains leads to the loss of many functions of Tat. CRD and BD are important for transactivation and induction of apoptosis. Additionally, CRD has also been shown to play a role in chemotactic properties of Tat [34] and BD has been reported to be important for the cellular entry via the protein transduction domain (PTD) although contradicting reports also exist for the role of PTD [32], [33], [35].

In addition to enhancing the safety profile by inactivating the transactivation potential, domain disruption of Tat also fulfilled the second objective of enhancing the antigenicity of Tat. The HTL-Tat proteins elicited significantly higher titer of antigen-specific antibodies as compared to the WT-Tat protein (Fig. 2A) and an enhanced T-cell proliferative response (Fig. 2B). Furthermore, the HTL-Tat proteins induced antibodies of higher avidity (Fig. 6A) that efficiently neutralized eTat (Fig. 6B). It is noteworthy that the HTL-Tat constructs were generated using subtype C Tat and several reports including ours has demonstrated C-Tat to be less toxic as compared to B-Tat. Subtype C Tat is less effective than B-Tat as a chemokine [34], less neurotoxic [36], [37] and possesses lower potential of transactivation [30]. Moreover, the antibodies generated against subtype C proteins cross-reacted strongly with heterologous Tat proteins. Given the safety advantage, subtype C Tat may be a better choice for Tat vaccine design.

Clade-specific variations and the emergence of immune escape variants pose a significant challenge to vaccine design. Antibody responses, therefore, must be broadly cross-reactive and tolerant to amino acid variations. Since NTE of Tat is the most immunodominant epitope of Tat, several peptide vaccines have been evaluated using this epitope [38], [39]. However, given that NTE is moderately conserved and subtype-specific variations are evident in this epitope, it becomes imperative that immune responses to multiple epitopes are elicited to counter the emergence of escape variants. Indeed, a recent Tat-epitope vaccine trial, despite inducing epitope-specific antibodies, had limited success in inhibiting viral rebound following anti-retroviral therapy (ART) cessation [40]. In this regard, it is noteworthy that the HTL-Tat proteins retain several antibody epitopes and upon immunization elicit a strong antibody response to multiple epitopes. Importantly, the magnitude of the immune response to the cryptic exon-2 epitope was comparable in magnitude to that of the dominant NTE suggesting a strong T-helper function. Interestingly, studies by us and other groups have shown that Tat DNA immunizations elicit a broader immune response targeting several epitopes [41], [42], whereas in protein immunizations, NTE is recognized as the most immunodominant epitope [42]–[44]. Therefore, it may be envisioned that a combined approach of genetic and protein immunization could lead to the activation of a strong cell-mediated as well as broad humoral immune responses.

Previous studies have suggested that Tat protein alters the Th1/Th2 profile [3], [45]. In mice, the isotypes IgG1 and IgG2a are representative of Th2 and Th1 respectively. Therefore, we evaluated the isotype profile of anti-Tat antibodies in immunized mice and found that IgG1 (Th2) was more prevalent as compared to IgG2a (Th1). One possible explanation for this could be the genetic predisposition of BALB/c mice to Th2. Also, the booster immunizations were carried out with incomplete Freund's adjuvant, which may influence the antibody response towards Th2. Despite these factors, HTL-Tat proteins not only enhanced the IgG1 response, but also increased IgG2a significantly as compared to the response elicited by WT-Tat (Fig. 5). These data suggest that HTL-Tat protein can enhance both Th1 and Th2 responses. Whether this phenotype is observable in other mouse strains such a C57BL/6J, which is Th1 biased, is being explored. Additionally, universal HTL-epitopes have been shown to enhance the cytotoxic T-lymphocyte (CTL) response, whether a robust CTL response is elicited along with the humoral response is an active area of our research.

The data presented here do not ascertain the helper nature of the Pol-HTL, as the epitope was grafted only in conjunction with the PADRE epitope and not tested independently. It is possible that in the context of Tat, only the PADRE epitope is functional in all the four HTL-engineered Tat proteins. The function of the Pol-HTL remains to be established in the appropriate context. Of note, the grafting of the PADRE epitope generated identical immune profile regardless of the Tat domain that was disrupted (PADRE-CRD and PADRE-BD). By inserting the HTL-epitopes between C30 and S31, we inadvertently disrupted a previously described epitope in CRD [21]. We demonstrated that the IgG antibodies in human sera reacting with the CRD epitope efficiently neutralized exogenous Tat. Grafting of the HTL between C22 and N23, instead of C30 and S31, should be appropriate to maintain the CRD B-cell epitope intact, yet abrogate Tat functions. Likewise, a B-cell epitope in BD was identified by human sera with an efficient Tat-neutralizing potential [46]. Our data demonstrated that the disruption of a single Tat domain, BD or CRD, is sufficient to enhance the safety profile of Tat (Fig. 1B) as well as augment its immunogenicity (Figs. 2 and 3B). Grafting of the HTL epitopes between C22 and N23 residues in the CRD therefore could be the best option to retain all the identified B-cell epitopes intact in Tat.

In summary, we have demonstrated a novel molecular strategy to enhance the immunogenic as well as the safety profiles of Tat by grafting universal T-helper epitopes into two domains that are critical for various biological functions of the viral regulatory protein. The strategy described here could be of significance for Tat-vaccine development.

## Supporting Information

S1 Fig **Lymphoproliferation assay.** Splenocytes isolated from the immunized mice 14 days following the final booster immunization were stained with 2.5 µM CFSE and incubated for 5 days in the presence of the Tat-peptides at a final concentration of 5 µg/ml. The cells were stained with anti-CD4-PE antibody and the proliferation was scored as percent CFSE-low cells. The x-axis represents CFSE-intensity and the y-axis to CD4-PE fluorescence intensity. The data are representative of two independent experiments.
(TIF)

S2 Fig **Epitope mapping.** The antisera collected from three of the four HTL-Tat immunized mice were diluted 1000-fold and used in the pepscan analysis. The schematic representation of the HTL-Tat constructs with the location of the peptides aligned with the protein frame on the left side. The dark bars (peptides 1-9) represent the sequences of the unmodified Tat protein. The gray bars (peptides A-H) represent the amino acid sequences generated due to the grafting of the HTL-epitopes in Tat. The mean absorbance + SD values are plotted on the x-axis with the corresponding peptides on the y-axis.
(TIFF)

S1 Table **Primers used for the construction of the HTL-Tat expression vectors.** The Tat domains targeted, the epitopes grafted and the primer numbers and sequences in the 5′ to 3′ orientation are presented. The restriction enzymes sites engineered into the primers are highlighted with bold fonts. The asterisk in the primer sequences represents the junction between two adjacent domains of Tat.
(XLSX)

S2 Table **The panel of peptides used for epitope mapping.** The peptides 1 through 9 span the full-length of subtype C Tat. The peptides A through H correspond to the sequences generated following the HTL-epitope insertion. The HTL-epitope sequences are highlighted with bold fonts.
(DOCX)
