# Supplementary figures and images for: The Grafting of Universal T-Helper Epitopes Enhances Immunogenicity of HIV-1 Tat Concurrently Improving Its Safety Profile
Source: PLoS One. 2014 Dec 22;9(12):e114155. doi: 10.1371/journal.pone.0114155 (PMC4273983; doi:10.1371/journal.pone.0114155)

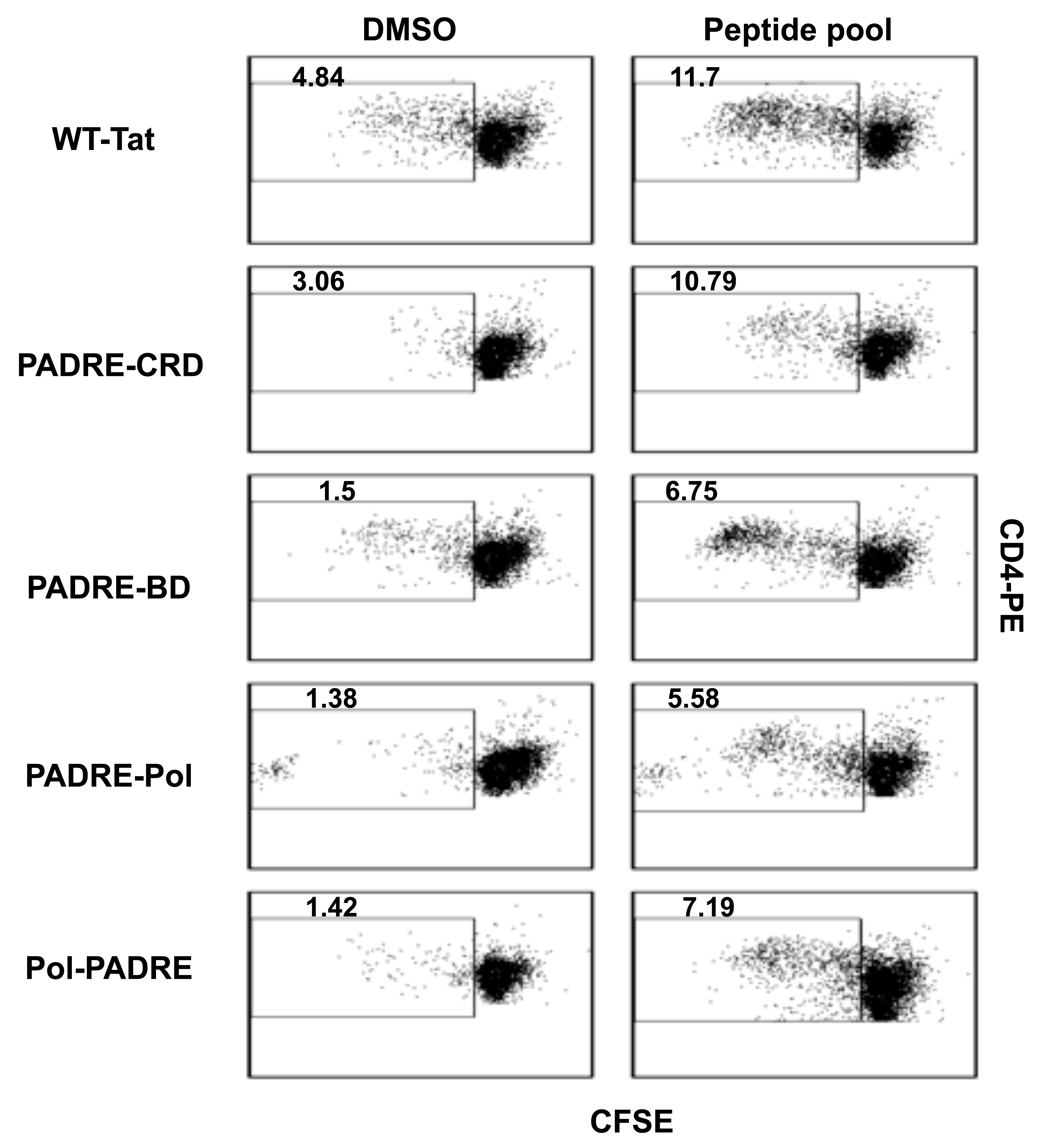

Supplement: S1 Fig — Lymphoproliferation assay. Splenocytes isolated from the immunized mice 14 days following the final booster immunization were stained with 2.5 µM CFSE and incubated for 5 days in the presence of the Tat-peptides at a final concentration of 5 µg/ml. The cells were stained with anti-CD4-PE antibody and the proliferation was scored as percent CFSE-low cells. The x-axis represents CFSE-intensity and the y-axis to CD4-PE fluorescence intensity. The data are representative of two independent experiments. (TIF) [file pone.0114155.s001.tif]

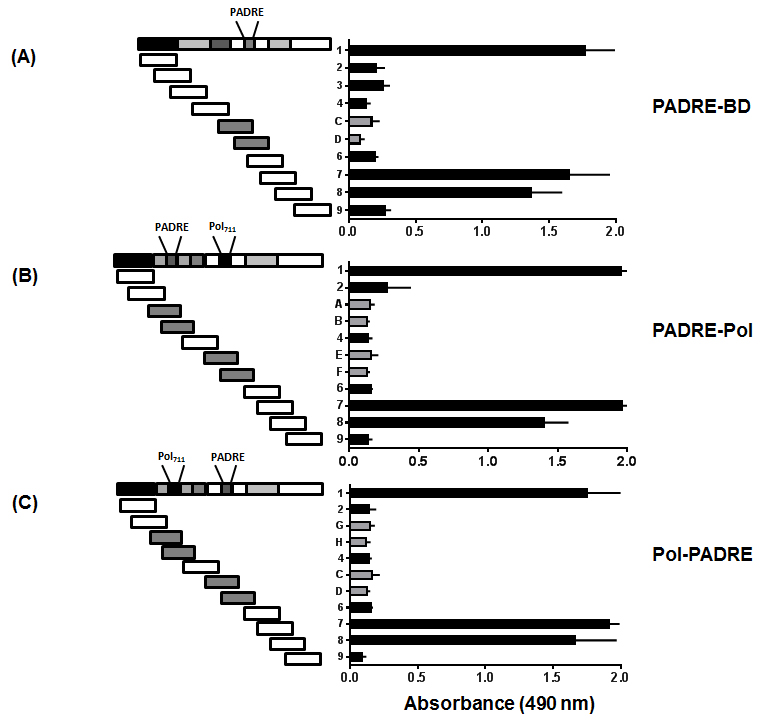

Supplement: S2 Fig — Epitope mapping. The antisera collected from three of the four HTL-Tat immunized mice were diluted 1000-fold and used in the pepscan analysis. The schematic representation of the HTL-Tat constructs with the location of the peptides aligned with the protein frame on the left side. The dark bars (peptides 1-9) represent the sequences of the unmodified Tat protein. The gray bars (peptides A-H) represent the amino acid sequences generated due to the grafting of the HTL-epitopes in Tat. The mean absorbance + SD values are plotted on the x-axis with the corresponding peptides on the y-axis. (TIFF) [file pone.0114155.s002.tiff]
