# Supplementary material for: The Grafting of Universal T-Helper Epitopes Enhances Immunogenicity of HIV-1 Tat Concurrently Improving Its Safety Profile
Source: PLoS One. 2014 Dec 22;9(12):e114155. doi: 10.1371/journal.pone.0114155 (PMC4273983; doi:10.1371/journal.pone.0114155)
Supplement: S2 Table — The panel of peptides used for epitope mapping. The peptides 1 through 9 span the full-length of subtype C Tat. The peptides A through H correspond to the sequences generated following the HTL-epitope insertion. The HTL-epitope sequences are highlighted with bold fonts. (DOCX) [file pone.0114155.s004.docx]

| **Peptide** | **Sequence** |
| --- | --- |
| **1** | MEPVDPNLEPWNHPGSQPKT |
| **2** | WNHPGSQPKTACNNCYCKRC |
| **3** | ACNNCYCKRCSYHCLVCFQK |
| **4** | SYHCLVCFQKKGLGISYGRK |
| **5** | KGLGISYGRKKRRQRRSAPP |
| **6** | KRRQRRSAPPSSEDHQNLISKQ |
| **7** | SSEDHQNLISKQPLPRTQGD |
| **8** | KQPLPRTQGDPTGSEESKKK |
| **9** | PTGSEESKKKVESKTETDPFD |
| **A** | ACNNCYSKHC***AKFVAAWTLKAAA*** |
| **B** | ***AKFVAAWTLKAAA***SYHCLVCFQT/K |
| **C** | GLGISYGRKK***AKFVAAWTLKAAA*** |
| **D** | ***AKFVAAWTLAAA***RRQRRSAPPS |
| **E** | ACNNCYCKRC***EKVYLAWVPAHKGIG*** |
| **F** | ***EKVYLAWVPAHKGIG***SYHCLVCFQT/K |
| **G** | GLGIC/GYGRKK***EKVYLAWVPAHKGIG*** |
| **H** | ***EKVYLAWVPAHKGIG***RRQRRSAPPS |
